# *Lyz2*-Cre-Mediated Genetic Deletion of *Septin7* Reveals a Role of Septins in Macrophage Cytokinesis and *Kras*-Driven Tumorigenesis

**DOI:** 10.3389/fcell.2021.795798

**Published:** 2022-01-06

**Authors:** Manoj B. Menon, Tatiana Yakovleva, Natalia Ronkina, Abdulhadi Suwandi, Ivan Odak, Sonam Dhamija, Inga Sandrock, Florian Hansmann, Wolfgang Baumgärtner, Reinhold Förster, Alexey Kotlyarov, Matthias Gaestel

**Affiliations:** ^1^ Institute of Cell Biochemistry, Hannover Medical School, Hannover, Germany; ^2^ Kusuma School of Biological Sciences, Indian Institute of Technology Delhi, New Delhi, India; ^3^ Institute of Immunology, Hannover Medical School, Hannover, Germany; ^4^ Institute of Genomics and Integrative Biology (CSIR-IGIB), New Delhi, India; ^5^ Institute of Pathology, Stiftung Tierärztliche Hochschule, Hannover, Germany; ^6^ Institute of Veterinary Pathology, Veterinary Faculty of Leipzig University, Leipzig, Germany

**Keywords:** septins, septin7, Lyz2-Cre, phagocytosis, myeloid cells, tumor model, Kras-G12D

## Abstract

By crossing *septin7*-floxed mice with *Lyz2*-Cre mice carrying the Cre recombinase inserted in the Lysozyme-M (*Lyz2*) gene locus we aimed the specific deletion of septin7 in myeloid cells, such as monocytes, macrophages and granulocytes. *Septin7* ^*flox/flox*^:*Lyz2-*Cre mice show no alterations in the myeloid compartment. *Septin7*-deleted macrophages (BMDMs) were isolated and analyzed. The lack of Septin7 expression was confirmed and a constitutive double-nucleation was detected in Septin7-deficient BMDMs indicating a defect in macrophage cytokinesis. However, phagocytic function of macrophages as judged by uptake of labelled *E. coli* particles and LPS-stimulated macrophage activation as judged by induction of TNF mRNA expression and TNF secretion were not compromised. In addition to myeloid cells, Lyz2-Cre is also active in type II pneumocytes (AT2 cells). We monitored lung adenocarcinoma formation in these mice by crossing them with the conditional knock-in *Kras*-LSL-G12D allele. Interestingly, we found that control mice without septin7 depletion die after 3–5 weeks, while the Septin7-deficient animals survived 11 weeks or even longer. Control mice sacrificed in the age of 4 weeks display a bronchiolo-alveolar hyperplasia with multiple adenomas, whereas the Septin7-deficient animals of the same age are normal or show only a weak multifocal brochiolo-alveolar hyperplasia. Our findings indicate an essential role of Septin7 in macrophage cytokinesis but not in macrophage function. Furthermore, septin7 seems absolutely essential for oncogenic *Kras*-driven lung tumorigenesis making it a potential target for anti-tumor interventions.

## Introduction

Septins, a conserved family of filament forming GTPases, build heteropolymeric higher-order structures and participate in diverse cellular processes, and are being widely accepted as the fourth component of eukaryotic cytoskeleton (Mostowy and Cossart, 2012). Originally discovered as genes required for cytokinesis in budding yeast, septins associate with mitotic spindle, contractile ring and midbody and participate in cytokinesis of metazoan cells, (Hartwell et al., 1970; Fededa and Gerlich, 2012; Green et al., 2012; Karasmanis et al., 2019; Chen et al., 2021). Non-canonical septin functions in mammalian cells range from neuronal morphogenesis to control of bacterial and viral infections (Bridges and Gladfelter, 2015; Van Ngo and Mostowy, 2019; Robertin and Mostowy, 2020).

Mammalian cells express 13 septin gene products with partially redundant functions. Depletion of one of the major subunits, such as the pivotal subunit Septin7 affects multiple steps in mitosis and cytokinesis (Kinoshita et al., 1997; Estey et al., 2010; Sellin et al., 2011; Karasmanis et al., 2019; Chen et al., 2021). Deletion of *Septin7* gene is associated with the co-depletion of the core septin subunits making it a pan-septin depletion model (Menon et al., 2014). *Septin7* ^*−/−*^ fibroblasts display cytokinetic failure and undergo obligate multinucleation (Menon et al., 2014). While *Septin7* ^*−/−*^ embryos fail to gastrulate, *Septin7*-deletion in lymphoid cells does not have an impact on T and B-lymphocyte development. However, *Septin7*-deleted CD8^+^ T cells display cytokinetic failure upon cytokine stimulation in the absence of antigen-presenting cells (Menon et al., 2014; Mujal et al., 2016). Furthermore, siRNA mediated Septin7 depletion in T-lymphocytes *in vitro* had no impact on lymphocyte proliferation, despite clear motility defects (Tooley et al., 2009). Septin7-deficient myeloid progenitors are capable of colony formation *in vitro* (Menon et al., 2014) and siRNA-mediated Septin7 depletion in myeloid K562 cell line had no impact on cell proliferation (Sellin et al., 2011). Septins assemble at the base of phagosomes in myeloid cells and siRNA mediated depletion of septin 2 and 11 in macrophages has been shown to suppress FcγR-mediated phagocytosis (Huang et al., 2008). Septins participate in the cell entry and pathogenicity of intracellular bacteria such as *Listeria* and *Shigella* (Mostowy et al., 2010; Van Ngo and Mostowy, 2019). While most of the studies investigating the role of septins in bacterial pathogenesis have been performed in non-phagocytic cells, an infection model in zebrafish has shown a role for macrophages and neutrophil septins in limiting *Shigella* infection (Mostowy et al., 2013). In the present study, we crossed the *Septin7* ^*flox*^ mice with the myeloid-specific *Lyz2*-Cre line (Clausen et al., 1999) to generate a tissue-specific *Septin7-*knockout (KO) mice and to investigate the role of septins in myeloid cells.

Adenocarcinoma, the major form of lung cancer, is associated with activating mutations in the oncogenic small GTPase KRAS (Cancer Genome Atlas Research Network, 2014). Most tumor cells located in lung periphery express surfactant protein C, indicating that they originate from alveolar type 2 (AT2) cells or from their progenitors (Desai et al., 2014). Since the *Lyz2* locus is active not only in myeloid lineage but also in AT2 cells (Desai et al., 2014), the Cre-Lyz2 knock-in strain could be used to analyze the effect of gene deletion in AT2 cells. Interestingly, AT2 proliferation is selectively induced by Cre-regulated oncogenic Kras-G12D *in vivo* (Jackson et al., 2001), efficiently generating multifocal, clonal adenomas in the lungs with replacement of almost the entire alveolar region and death of the animals within a month after birth (Desai et al., 2014). The generation and initial analyses of the *Septin7* whole-animal and hematopoietic-specific knockout models had revealed a cell-type specific role for septins in mammalian cytokinesis (Menon et al., 2014). These findings support a hypothesis that differential targeting of cytokinesis could form the basis for specific anti-proliferative therapies to target solid tumors, without impairing hematopoiesis that is less dependent on septins (Menon and Gaestel, 2015). Here, we present genetic evidence that septins are indispensable for Kras-induced lung tumorigenesis and thus septin targeting may be a feasible strategy of therapeutic intervention.

## Materials and Methods

### Generation of *Lyz2*-Cre Driven *Septin7* Conditional KO Mice

All mice experiments were conducted according to German and international guidelines and were in accordance with the ethical oversight by the local government for the administrative region of Lower Saxony (permit 17/2593), Germany. *Septin7* ^flox/flox^ mice (*Sept7* ^*tm1Mgl*^) targeting the exon 4 of Septin7 gene was reported previously (Menon et al., 2014). For the generation of myeloid specific knockout animals, *Septin7* homozygous floxed mice (back-crossed for six generations with C57Bl/6J) were crossed with *B6- Lyz2* ^*tm1(cre)Ifo*^ deletor line (Clausen et al., 1999). For the generation of the Ras-induced tumor model the *Sept7* ^*flox/flox*^ *:Lyz2-Cre* strain was crossed with *B6.129S4-Kras* ^*tm4Tyj*^ animals (Jackson et al., 2001). In animal experiments, age and sex matched, Cre-expressing *Septin7* wt/flox (heterozygous control, *n* = 18) and flox/flox (homozygous floxed, *n* = 17) mice were compared. Mice were monitored daily for survival.

### DNA Isolation and Genotyping

Tail biopsies and BM samples were overnight digested at 53°C in lysis buffer [50 mM Tris-Cl (pH 8.0), 100 mM EDTA, 100 mM NaCl and 1% SDS] containing proteinase-K (0.5 mg/ml) (Roche). Proteins were salted out with extra NaCl. DNA was precipitated with isopropanol, washed with 70% ethanol and dissolved in water. Genotyping PCRs were performed with Top Taq DNA polymerase (Qiagen) with extra Mg^2+^ under standard conditions with annealing temperature at 53°C. The primers used for *Septin7* and *Cre* genotyping were described previously (Menon et al., 2014). Lyz2-Cre locus was genotyped using primers described in the Jackson laboratory Genotyping protocol (#28518) for the strain using standard PCR conditions. For genotyping *Kras*, the following primers were used: Kras-y117: 5’-CTA​GCC​ACC​ATG​GCT​TGA​GT-3’; Kras-y118: 5’-ATG​TCT​TTC​CCC​AGC​ACA​GT-3’; Kras-y116: TCC​GAA​TTC​AGT​GAC​TAC​AGA​TG-3’. For *Kras* wildtype, y117/y118 primer pairs generated a PCR product of 450-bp. For *Kras LSL* allele y117/y116 primer pairs generated a PCR product of 327-bp (Thakkar et al., 2018) PCR reactions were separated on 2% agarose gels and images acquired using INTAS Gel documentation system.

### BMDM Generation and Treatments

To obtain bone marrow-derived macrophages (BMDMs), femurs of 6–8 weeks-old mice were flushed and plated on one 10 cm plate with 100 ng/ml M-CSF (Wyeth/Pfizer) in DMEM supplemented with 10% FCS, antibiotics and 1× non-essential amino-acids. The next day, non-adherent floaters were transferred to a new 10 cm plate and new medium containing M-CSF was added to the first plate. Medium was renewed on both plates every 3–4 days. Cells were scraped and seeded for experiments between 10 and 14 days after initial plating. For experimental treatments, cells were seeded in the same growth medium without MCSF. For stimulation, BMDMs were treated with LPS (Sigma, *E. coli* O127:B8, 1 μg/ml) for indicated time-periods before cells and/or supernatants were used for further analyses.

For monitoring phagocytosis, the cells were treated with BODIPY™ FL conjugated *E. coli* (K-12 strain) Bioparticles (Molecular Probes, Cat#E2864) for 75 min. Immediately after the incubation, cells were cooled on ice, were 3× washed with ice-cold PBS and were fixed and stained with Septin7 antibodies and DAPI as described below. For semi-quantitative measurement of phagocytosis BMDMs were treated with Fluorescein-labeled *Escherichia coli* K-12 Bioparticles (Vybrant^™^ Phagocytosis Assay Kit, Molecular Probes, Cat#V-6694) for 75 min. After incubation, cells were immediately cooled on ice and trypan blue was added and incubated for 1 min to extinguish fluorescence of non-internalized bacteria. The cells were washed 3× with ice-cold PBS, fixed with 4% PFA and stained with DAPI as described below. The images were acquired on Cytation 1 (BioTek) at 40× magnification in eight fields of view (FOV) per samples using BioTek Gen5™ Software. Quantification of mean fluorescence intensity (MFI) was determined using Cellular Analysis tools from BioTek Gen5™ Software. DAPI staining was defined as primary mask to identify the cells. Furthermore, the secondary mask was defined from GFP channel by expanding the primary mask to 20 µm that cover the perinuclear region. Green MFI in that area were measured. The results are defined as the mean of green fluorescence intensity per cells per FOV. For monitoring the efficiency of Septin7 depletion, control cells were fixed and stained for Septin7 (Alexa fluor-555 channel) and images were acquired and processed as above. DAPI staining was defined as primary mask to identify the cells and the standard RFP channel was used to detect Septin7. RFP mean fluorescence intensity (MFI) were measured and a minimum threshold RFP signal in the Septin7 positive cells were defined. The number of cells with red fluorescence intensity higher than the threshold were counted, and then the percentage of positive cells to total cells (DAPI positive cells) were calculated. The results are the mean of % RFP positive cells per FOV.

### Antibodies and Reagents

Primary antibodies used were: Septin7 (#JP18991, IBL International), EF2 (sc-13004-R, Santa Cruz Biotech) and Phospho-p38 MAPK (#9211, Cell Signaling Technology). Anti-rabbit Alexa fluor-555 (#A31572), anti-rabbit Alexa fluor-488 (cat# A21206) and anti-mouse Alexa fluor-546 -dye labeled secondary antibodies, tetramethyl rhodamine-conjugated WGA (#W849) and Alexa fluor-647-conjugated phalloidin (#A22287) were from Invitrogen. DAPI for DNA staining was from Carl Roth (#6335.1). HRP-labeled anti-rabbit (#111035-003) secondary antibodies for immunoblots were from Dianova.

### Western Immunoblotting

Cells were lysed directly in SDS gel loading dye and western blotting was performed as previously described using gradient SDS-PAGE gels (Menon et al., 2010).

### Immunofluorescence Staining

BMDMs were grown on glass coverslips and fixed with 4% paraformaldehyde (PFA) in PBS. Fixation was performed for 2–5 min at room temperature (RT) followed by 20 min at 4°C and were permeabilized with 0.25% Triton X-100–PBS for 30 min at RT. Blocking was done using 4% bovine serum albumin (BSA) for 1 h at 4°C. Primary antibodies were used at a 1:50 to 1:200 dilution in 1% BSA–PBS for 1–2 h. Secondary antibodies or Alexa Fluor 647-conjugated phalloidin/tetramethy rhodamine conjugated WGA was used at a 1:500 dilution in 1% BSA–PBS. Slides were mounted with ROTI^®^Mount (Carl-Roth) after staining with DAPI. Imaging was performed using a Leica TCS SP2 confocal microscope with standard settings. For phagocytosis assays, images were acquired using a standard fluorescence microscope or the Cytation 1 imaging multimode reader (Biotek).

### Flow Cytometry Analysis

For immunophenotyping analysis of bone marrow, cells were isolated, RBC lysed (Pharmlyse, BD Biosciences) and analyzed for surface staining with Gr1-PE (clone-RB6-8CS), Sca1-PE (D7), ckit-APC (2B8; BD Biosciences), CD11b-APC (M1/70; eBiosciences), CD11b-PE (M1/70.15; Immunotools)and F4/80-PE (BM8; eBiosciences) as described previously (Mingay et al., 2018). In short, Propidium-Iodide-negative cells were gated and probed for c-Kit-APC/Sca1-PE double positive, CD11b-APC/F4/80-PE double positive (monocytes) and CD11b-APC/Gr1-PE double positive (granulocytes) cells as shown in the representative gating scheme (Supplementary Figure S1). BMDMs were scraped out, pelleted and resuspended in PBS-2mM EDTA before staining. Samples were analyzed using a FACSCalibur Flow-cytometer (BD Biosciences).

For Septin7 staining, nucleated BM cells were fixed with 3× by volume PFA (4%) at RT for 30 min. Washed and resuspended in PBS and absolute methanol was added to 90% concentration final with constant mixing. The methanol permeabilization was continued for 30 min on ice. After 2× PBS wash cells were resuspended in 4% BSA-PBS and blocked at 4°C for 30 min. Cells were stained with primary Septin7 antibodies (1:100 in 1%BSA-PBS) at RT for 30 min. After 1× PBS wash, samples were resuspended in Alexa fluor-488-labelled secondary antibody dilution (1:500 diluted in 1% BSA-PBS) and incubated for additional 30 min before PBS wash and analysis in Accuri- C6 flow cytometer.

### Gene Expression Analyses by Real-Time qPCR and ELISA

RNA was isolated using the NucleoSpin RNA extraction kit (Macherey and Nagel) according to the manufacturer’s instructions. cDNA was synthesized with the first strand cDNA synthesis kit (Fermentas/Thermo) using random hexamer primers. qRT-PCRs were run on a Rotor-Gene-Q (Qiagen) device using 2× SYBR-Green SensiFast mixes (no ROX, Bioline) and the gene expression for *Tnfα* and *Nfkbiα* were normalized to *Gapdh* and presented. Primers used are *Tnfα*-fwd- 5’-TGC​CTA​TGT​CTC​AGC​CTC​TTC-3’, *Tnfα* -rev- 5’- GAG​GCC​ATT​TGG​GAA​CTT​CT -3’, *Gapdh*-fwd- 5’- CAT​GGC​CTT​CCG​TGT​TCC​TA- 3’, *Gapdh*-rev-“CCTGCTTCACCACCTTCTTGAT”, *Nfkbiα* -fwd-5’- GAC​GCA​GAC​CTG​CAC​ACC​CC- 3’ and *Nfkbiα*-rev-5’-TGGAGGGCTGTCCGGCCATT-3’. BMDM supernatants were collected and quantification of murine TNFα by ELISA was performed as described previously using commercial kits (Tiedje et al., 2012).

### Histopathology

Lungs were harvested and inflation fixed with 10% neutral buffered formalin. Lungs were trimmed at different levels and embedded in paraffin wax. For histological examinations 2–3 µm thick sections were prepared and stained with hematoxylin and eosin.

### Statistical Analyses

**FIGURE 1:**
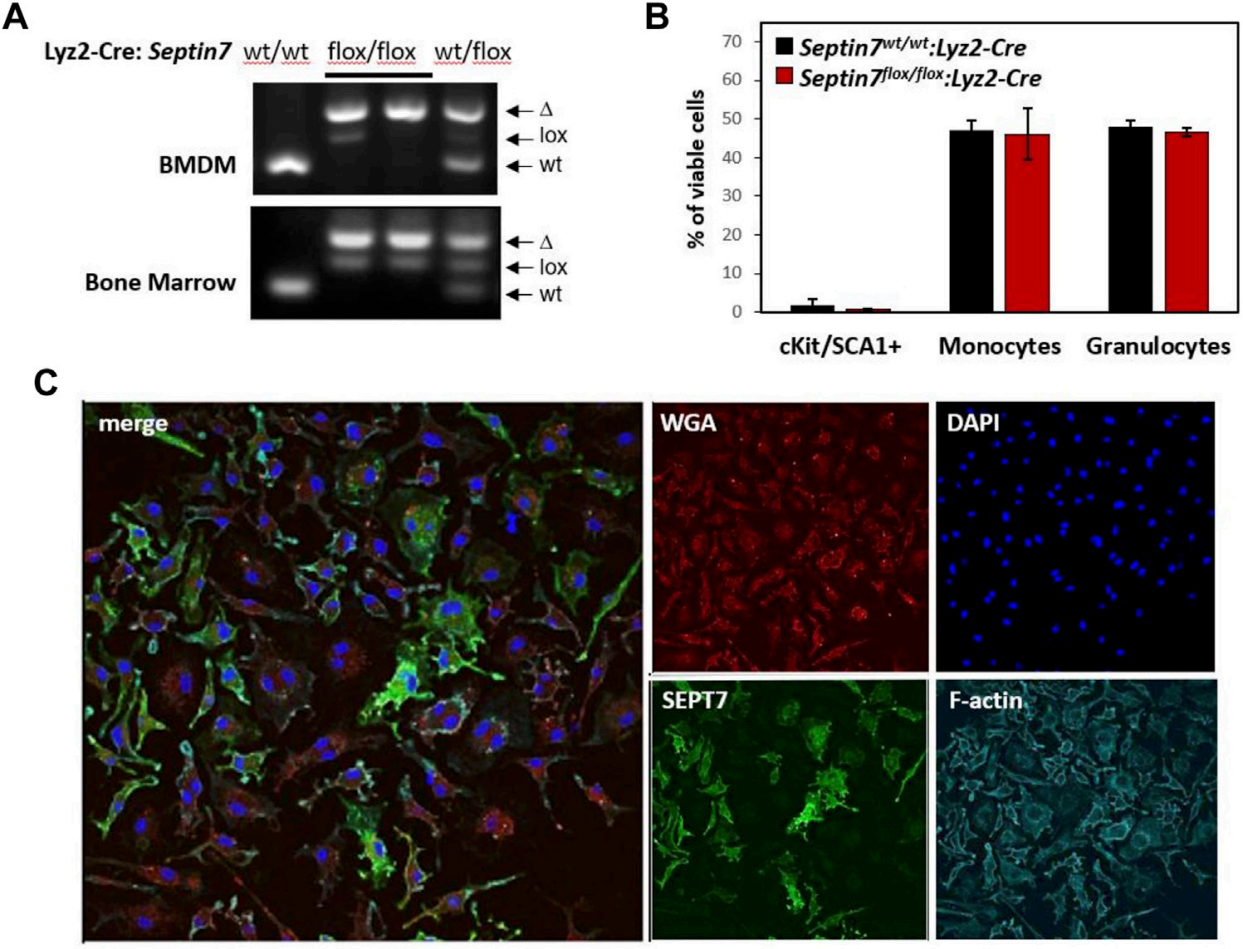
Effect of myeloid Septin7-deletion in myelopoiesis and BMDM proliferation. **(A)** Septin7-genotye analyses of bone marrow (BM) cells and BM-derived macrophages (BMDM) from the myeloid specific Septin7-KO mice show efficient deletion of the floxed allele. **(B)** Cell surface marker analysis of BM cells from *Septin7* ^*flox/flox*^ *:Lyz2-Cre* and *Septin7* ^*wt/wt*^ *:Lyz2-Cre* reveal no significant differences (*n* = 3 mice each, *p* value > 0.3) in stem/progenitors (cKit^+^ and Sca1^+^), monocytes (CD11b^+^ and F4/80^+^) and granulocyte (Gr1^+^ and CD11b^+^) populations. **(C)** Immunofluorescence analysis showing obligatory binucleation of *Septin7*-KO BMDMs. DAPI is used for nuclear staining, WGA and Phalloidin (staining F-actin) are shown as counter stains. Septin7-negative cells display more than one nucleus, whereas Septin7-positive cells (stained green) display only one.

**FIGURE 2:**
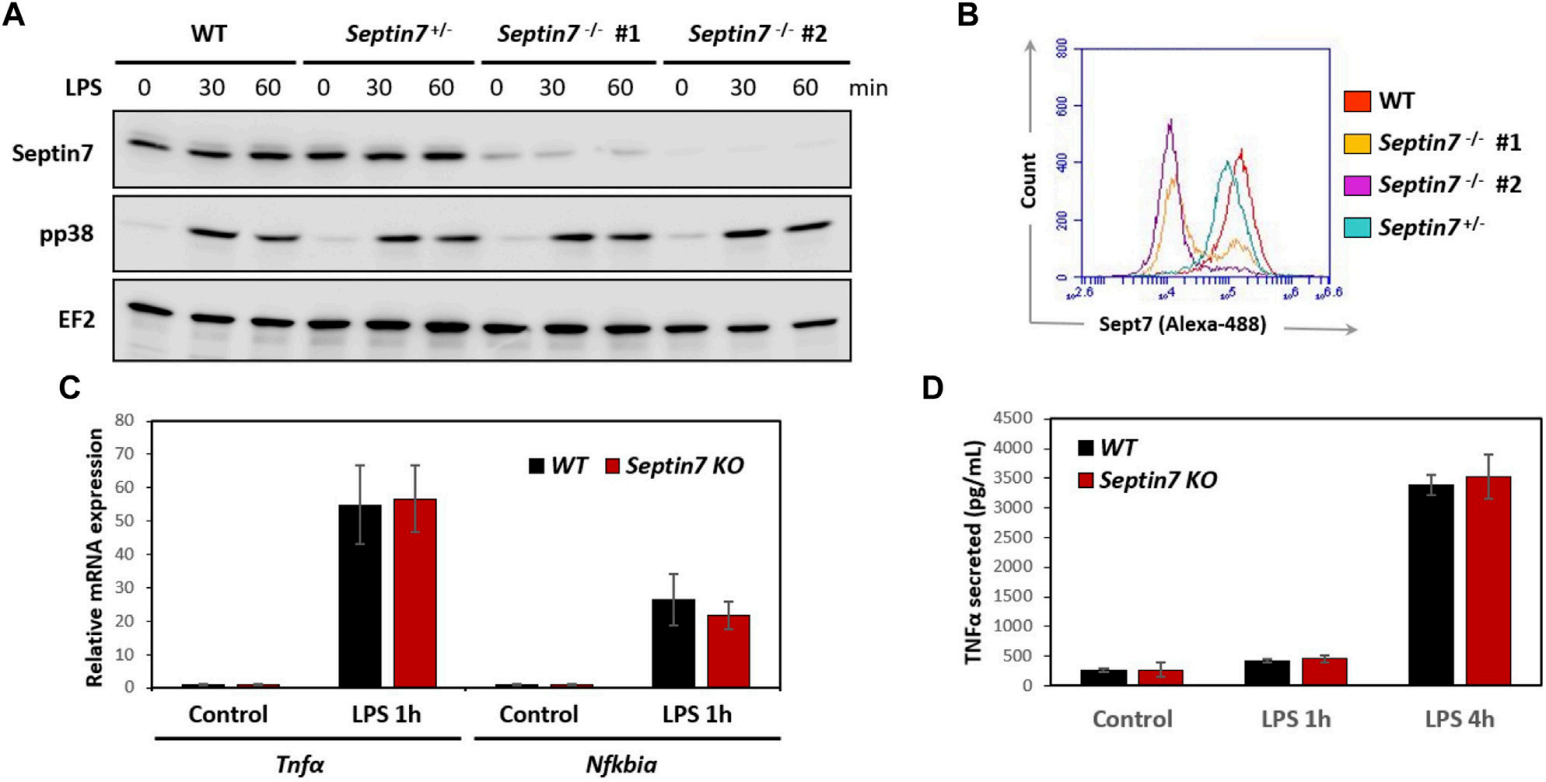
Septin7 KO BMDMs respond normally to LPS stimulation. **(A,B)** Immunoblot analyses of LPS-stimulated BMDM lysates **(A)** and Flow-cytometry **(B)** show efficient depletion of Septin7 in two homozygous *Septin7*-KO cells. However, p38 activation is indistinguishable between the KO and control cells **(A)**. EF2 is shown as loading control. **(C)** LPS-induced expression of *Tnfα* and *Nfkbiα* are not significantly affected upon *Septin7*-deletion in BMDMs (*n* = 4 independent treated culture wells, *p*-values > 0.3 for 1 h LPS-stimulated samples). **(D)** LPS-induced secretion of Tnfα quantified by ELISA reveal no significant difference between *Septin7*-KO and *Septin7*-Wt (WT) BMDMs (*n* = 4 independent treated culture wells, *p*-values > 0.3 for 1 and 4 h LPS-treated).

Students 2-tailed *t*-tests was used to check the statistical significance of differences between Septin7-wt and KO myeloid cells (in Figure 1B, Figures 2C,D, *p*-values non-significant > 0.3). For the animal experiments, *p*-values were derived by the Kaplan-Meier log-rank (Mantel-Cox) test using GraphPad Prism 8 software. The survival curves are significantly different with a *χ* ^2^ = 36.29 resulting in a *p* value < 0.0001.

## Results

### Deletion of *Septin7* Does Not Alter the Myeloid Compartment, But Leads to Cytokinesis Failure in Macrophages *In Vitro*

By *in vitro* deletion of *Septin7* in hematopoietic cells, we had previously shown that *Septin7*-deficient myeloid progenitors can form colonies in a semi-solid medium (Menon et al., 2014). To further investigate the *in vivo* consequences of *Septin7* deletion in myeloid cells, we crossed the *Septin7*-floxed mice with the *Lyz2*-Cre deleter line (Clausen et al., 1999) to generate a myeloid-specific *Septin7*-KO. *Septin7* ^*flox/flox*^ *:Lyz2-Cre* animals developed normally without any perceived abnormalities. Efficient Cre-mediated recombination was detectable in the bone marrow (BM) of the heterozygous and homozygous floxed mice in the presence of *Lyz2*-Cre as indicated by the genotyping data (Figure 1A). Residual amounts of the floxed allele were detected, as expected from the presence of additional non-myeloid cells in the BM. Flow-cytometric analysis for the myeloid lineage cells revealed no significant difference between the wild-type and *Septin7*-KO mice BM, indicating septin-independent development of myeloid lineage cells (Figure 1B).

In regard to the cell-type specificity of septin-dependent cytokinesis, a recent hypothesis is the requirement of septins specifically for the proliferation of adherent cell-types (Sellin et al., 2011). To verify this hypothesis, we generated adherent BM-derived macrophages (BMDMs) from the *Septin7*-KO mice. As indicated by the genotyping data, the BMDMs displayed almost complete deletion of the *Septin7*-floxed allele (Figure 1A). However, microscopic analyses of Septin7 expression revealed significant heterogeneity in the cells even after 10 days of BMDM differentiation showing a mosaic of Septin7-depleted and Septin7 positive cells (Figure 1C). Interestingly, cells without Septin7 expression were almost exclusively double-nucleated, establishing a role for septins in BMDM cytokinesis.

### *Septin7*-Deleted Macrophages are Functional, Despite Defective Cytokinesis

**FIGURE 3:**
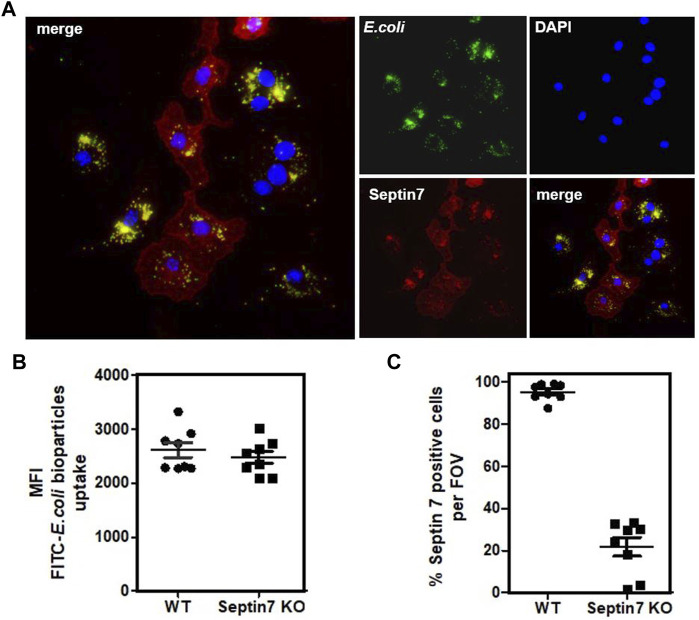
Septin7 KO BMDMs phagocytose bacterial particles. **(A)** BMDMs generated from *Septin7* ^*flox/flox*^ *:Lyz2-Cre* mice BM were subjected to phagocytosis assay with fluorescent labeled *E. coli* particles (Green) and were fixed and stained with Septin7 antibodies (Red) and DAPI (blue). Both Septin7-positive mono-nucleated cells and Septin7-deficient predominantly multinucleated BMDMs efficiently phagocytose the bacterial particles as indicated by their intracellular accumulation. **(B)** Semi-quantitative analysis of phagocytosed bacterial particles of WT and Septin 7 KO BMDMs—mean of green fluorescence intensity (MFI) of phagocytosed green-fluorescent particles is shown per cells per field of view. **(C)** Percentage of Septin7-positive cells quantified as detailed in methods section and shown as percentage per field of view (FOV).

Considering the cytokinetic defect observed in Septin7-deficient macrophages, we further investigated whether they are impaired in their functionality. Expression of myeloid markers including CD11b and F4/80 was comparable between control and Septin7^*−/−*^ BMDMs indicating normal differentiation *in vitro* (Supplementary Figure S2). Immunoblotting revealed strong depletion of Septin7 protein levels in the *Septin7* ^*flox/flox*^ *:Lyz2-Cre* BMDMs, which was consistent with the flow-cytometric analysis (Figures 2A,B). To understand the effects of Septin depletion of macrophage functions, we monitored the lipopolysaccharide (LPS) induced activation of BMDMs by analyzing downstream p38 MAPK phosphorylation. As expected, strong p38 activation was observed upon LPS-stimulation, but there were no differences between Septin7-depleted and wild-type cells (Figure 2A). Real-time qPCR analyses for the expression of mRNA of *Tnfα* (TNFα) and *Nfκbia* (IκBα), two downstream targets of LPS, also showed no significant changes upon *Septin7* deletion (Figure 2C). Moreover, the LPS-induced secretion of TNFα was also normal in Septin7-deficient BMDMs (Figure 2D). Since the alterations in septin cytoskeleton may have consequences in vesicular transport and processes involving the cell cortex, we also performed macrophage phagocytosis assays using labelled *E. coli* particles. Since Lyz2-Cre mediated deletion of Septin7 usually results in a mixed population of *Septin7*-positive and negative cells in the early stages, we used this model to monitor the role of septins in phagocytosis. As indicated by the images, the BMDMs efficiently phagocytosed the bacterial particles independent of Septin expression and multinucleation (Figure 3A). Semi-quantitative analysis using immunofluorescence microcopy showed no differences in phagocytosed bacterial particles between WT and Septin 7 KO BMDMs (Figure 3B). A control analysis of untreated cells revealed that majority of BMDMs from Septin7 KO were indeed depleted of Septin7 protein (Figure 3C).

In summary, while Septin7-depleted macrophages displayed cytokinetic failure leading to bi-nucleated and multinucleated cells, they were normal for the functions tested. It is interesting to note that fully differentiated and activated macrophage populations usually harbor significant amount of multinucleated cells (Pereira et al., 2018).

### *Septin7* in Tumor Cell Proliferation *In Vivo*

**FIGURE 4:**
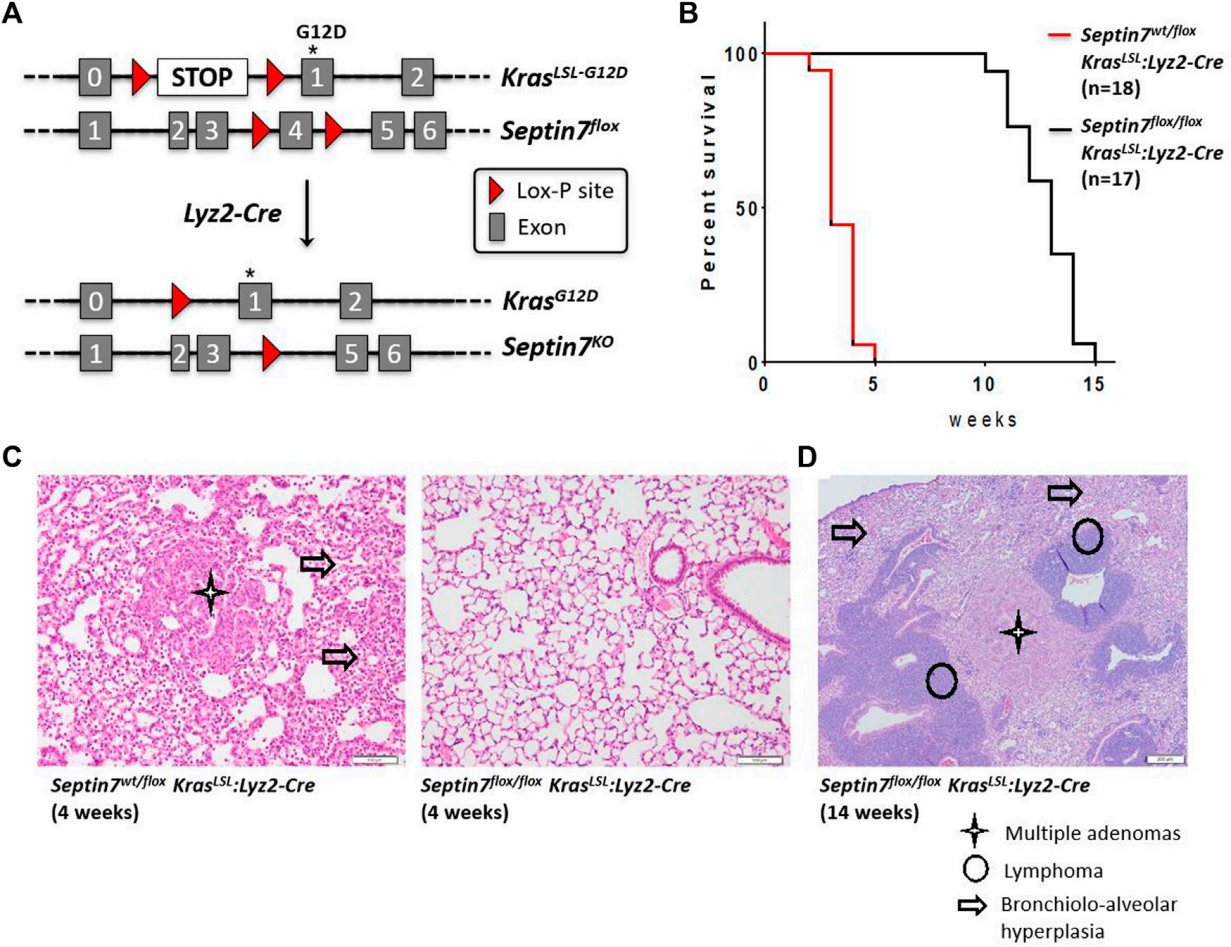
Septin7 is essential for Ras-induced lung tumor formation and lethality *in vivo*. **(A)** The gene targeting strategy employed for the generation of a Kras-G12D-induced lung tumor model to monitor the effects of Septin7-deficiency using the Lyz2-Cre deleter line. **(B)** Kaplan-Meier survival plots showing the essential role of Septin7 in lung cancer induced lethality in the Kras-induced tumor model. The survival curves are significantly different with a *χ* ^2^ = 36.29 resulting in a *p-*value < 0.0001. **(C)** Representative images of hematoxylin and eosin (H and E) stained lung sections from the mice of indicated genotype at 4 weeks after birth. Cancer associated tissue alterations visible are indicated (scale bar = 100 µm). **(D)** Lungs from Septin7-KO mice show late-onset adenomas and lymphomas as revealed in the standard histology H and E staining. Arrow = bronchiolo-alveolar hyperplasia, star = multiple adenomas and circle = T cells lymphoma (scale bar = 200 µm).

Lysozyme M (*Lyz2*)-Cre is often used as the deleter line of choice for gene deletion in myeloid lineages including monocytes, macrophages and granulocytes. However, the *Lyz2* locus is active not only in myeloid lineages but also in the lung alveolar type-2 (AT2) cells. Hence, the *Cre*-Lyz2 knock-in strain could nicely be used to analyze the effect of gene deletion in AT2 cells. AT2 cells, among other things are involved in the secretion of surfactants that prevents alveolar collapse. Analysis of the *Septin7* ^*flox/flox*^ *:Lyz2-Cre* mice revealed no defects in lung development (data not shown). To analyze the role of Septin7 in tumor development, we decided to establish a Septin7-dependent *in vivo* mouse model of lung adenocarcinoma formation, the major form of lung cancer associated with activating mutations in the *Kras* oncogene. We used Cre-Lyz2-mediated recombination to activate the conditional knock-in *Kras*-*LSL-G12D* allele (Jackson et al., 2001) in AT2 cells (Desai et al., 2014). In this model, AT2 proliferation is selectively induced by knock-in of oncogenic *Kras-G12D in vivo*, efficiently generating multifocal, clonal adenomas with replacement of almost the entire alveolar region and death of the animals within 1 month after birth. We genetically combined the knock-in of oncogenic *Kras* with the deletion of *Septin7* in AT2 cells *in vivo* by crossing mice to introduce the *Sept7* ^*flox*^-allele in addition to *Kras-LSL-G12D* and *Lyz2-Cre* alleles (Figure 4A). We then compared adenocarcinoma formation between *Kras-LSL-G12D:Lyz2-Cre*:*Sept7* ^*flox/flox*^ and *Kras*-LSL-G12D:Lyz2-Cre:*Sept7* ^*flox/wt*^. All animals analyzed were heterozygous for the *Kras-LSL-G12D* allele and littermates were used to avoid other variables. We detected a significant difference in survival time between the homozygous *Septin7*-KO and the control *Septin7* ^*+/−*^ mice. While control mice die between 3–5 weeks, the Septin7-deficient animals survived 11 weeks or even longer (Figure 4B). In parallel, lungs from both groups of animals were investigated using histopathology. The occurrence of bronchiolo-alveolar hyperplasia as well as adenomas was assessed semi-quantitatively by light microscopy. Analyses of control mice sacrificed at the age of 4 weeks displayed a bronchiolo-alveolar hyperplasia with multiple adenomas, whereas lungs of Septin7-deficient animals of the same age were unremarkable or showed only a weaker multifocal brochiolo-alveolar hyperplasia (Figures 4C,D and Supplementary Figure S4). This indicates an essential role of Septin7 in oncogenic Kras-induced lung tumorigenesis.

## Discussion

Our findings from the myeloid-specific *Septin7*-KO model indicate an essential role of *Septin7* in macrophage cytokinesis but not in macrophage functions. Furthermore, septin7 seems to be essential for Kras-driven tumor development in the lung making it a potential target for anti-tumor interventions. Even though it was a known fact that septins are required for the proliferation of several tumor-derived epithelial cell lines and Septin7-deficient fibroblasts undergo obligate multinucleation *in vitro*, this is the first report establishing a clear role for septins in tumorigenesis *in vivo*.

The *Lyz2-Cre*-mediated *Septin7* deletion was very efficient in the BM, but did not lead to perceivable defects in the development of monocytes and granulocytes (Figures 1A,B). However, *in vitro* differentiated BMDMs displayed clear double nucleation upon Septin7-depletion. Interestingly, this supports the hypothesis that adherent hematopoietic lineages and not the suspension cells require septins for completion of cytokinesis. It should be noted that the complete depletion of Septin7 in majority of the BMDMs was only achieved upon prolonged differentiation of BM cells with MCSF. While 1 week of MCSF treatment is used in standard BMDM generation protocols, much longer periods were required to achieve Septin7 deletion in majority of the population (Supplementary Figure S5). Despite the defects in cytokinesis, the *Septin7*-KO macrophages were not compromised in their functions including activation, gene expression, cytokine secretion and phagocytosis. Previous studies using knockdown approaches targeting Septins 2 and 11 in macrophage cell lines have shown a role for septins in phagosome assembly (Huang et al., 2008). Moreover, they observed a septin collar-like structure at the base of phagosomes during FcγR-mediated phagocytosis in macrophages and neutrophils. This discrepancy with our observations could be due to the differences in the experimental settings as the previous study specifically focused on FcγR-mediated phagocytosis of IgG-coated latex beads and we followed phagocytosis of bacteria by primary macrophages. A role for septins in bacterial and yeast infection or invasion has been established predominantly in non-phagocytic epithelial cells and the *Septin7* ^*flox/flox*^ *:Lyz2-Cre* mouse model will be an invaluable tool in further studies investigating the role of septins in infection biology using septin-depleted macrophages and neutrophils.

The protection of the Septin7-depleted mice from rapid-onset lung tumors and resultant lethality was clearly evident. However, *Septin7*-KO mice also eventually succumbed to the oncogene-induced lethality. We could detect adenomas similar to that of the 4 weeks-old control mice in *Septin7* ^*flox/flox*^ animals sacrificed at the age of 14 weeks. These “late” (slow growing) adenomas could result from cells adapted to *Septin7* ablation or could also arise from a minor cell population with Cre-driven recombined *Kras* but without Cre-driven *Septin7* deletion. Interestingly, adenomas in the lungs of *Septin7* ^*flox/flox*^ animals were accompanied with morphological distinct tumor foci (myeloma or lymphoma), which were not detected in the control animals. A possible explanation is that the growth of adenoma was inhibited in the absence of Septin7 and other cell types with activated *Kras* form tumors with a slower kinetics. It is to be noted that *Lyz2-Cre* expression is not restricted to myeloid cells and AT2 cells (Ye et al., 2003). Another possibility is that the appearance of this novel (non-adenoma) tumor cells is caused by *Kras* activation combined with genetic instability caused by the loss of Septin7. Interestingly, a recent multi-omics study correlated low SEPTIN7 expression levels and an intronic single-nucleotide polymorphism in the *SEPTIN7* gene with higher survival rates for long-term former smoking lung cancer patients (Shen et al., 2021). While the presence of Septin7 seems indispensable for oncogenic Ras-induced lung-adenocarcinoma formation, further investigations in the *Septin7* conditional knockout model is necessary to completely understand the role of septins in tumorigenesis.

## Data Availability

The raw data supporting the conclusions of this article will be made available by the authors, without undue reservation.

## Supplementary Material

Supplementary Figure S1 The gating scheme used for immunophenotyping of the bone-marrow. The simple gating scheme involving forward, and side-scatter based gating, followed by dead-cell exclusion by selecting propidium-iodide negative cells is shown with a representative example bone-marrow staining with the indicated antibody cocktails.

Supplementary Figure S2 Septin7-KO BMDMs have normal surface marker expression. Septin7-KO and wild-type BMDMs were surface stained for CD11b or F4/80 and were analysed by BD Accuri-C6 flow-cytometer.

Supplementary Figure S3 Isotype controls show specific staining of Septin7 in BMDMs. Additional controls for the flow-cytometry data presented in Figure 2B. An indirect flow-cytometry protocol is used with anti-Septin7 antibodies and anti-Rabbit Alexa Fluor-488 labelled secondary antibodies are 13 combined to detect septin. Here, staining with the secondary antibody alone is included which shows immunofluorescence intensities similar to that seen in the Septin7-KO cells.

Supplementary Figure S4 Higher magnification images showing the abnormalities observed in old Septin7 KO mice upon Ras-induced tumorigenesis. Representative higher magnification images (scale bar = 100 μm) of H&E stained sections from Septin7-KO mice (14 weeks old, shown in Figure 4D).

Supplementary Figure S5 Effect of longer BMDM culture on Septin7 depletion in floxed cells. BM cells from WT and Septin7-floxed mice were treated with MCSF for the indicated time-periods to generate BMDMs. The adherent cells were fixed and stained for Septin7, alpha-tubulin and nuclei (DAPI). The data clearly indicate significantly higher number of Septin7-depleted cells in 2 weeks differentiated compared to 1 week differentiated KO cells.
