# Supplementary figures and images for: Lyz2-Cre-Mediated Genetic Deletion of Septin7 Reveals a Role of Septins in Macrophage Cytokinesis and Kras-Driven Tumorigenesis
Source: Front Cell Dev Biol. 2022 Jan 6;9:795798. doi: 10.3389/fcell.2021.795798 (PMC8772882; doi:10.3389/fcell.2021.795798)

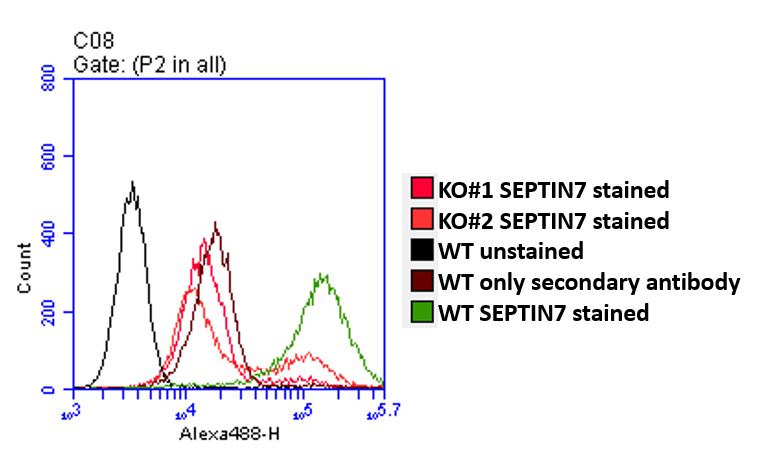

Supplement: Supplementary file 1 [file Image3.JPEG]

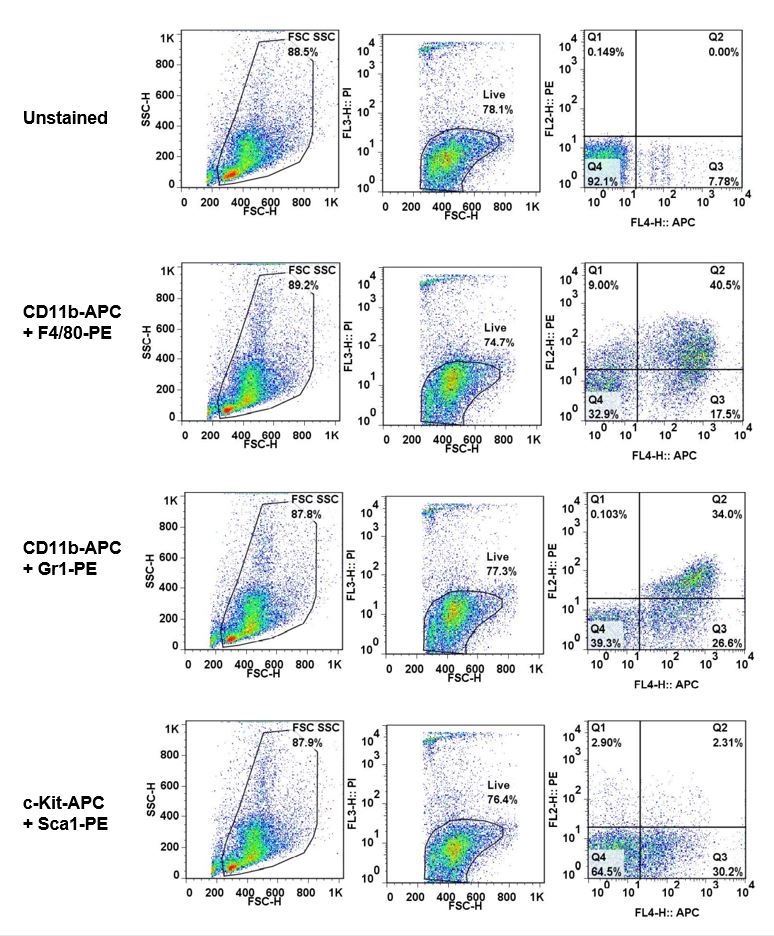

Supplement: Supplementary file 2 [file Image1.JPEG]

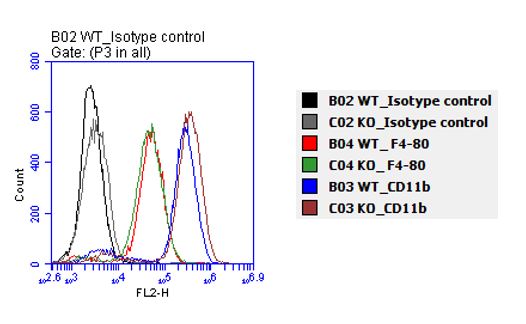

Supplement: Supplementary file 3 [file Image2.JPEG]

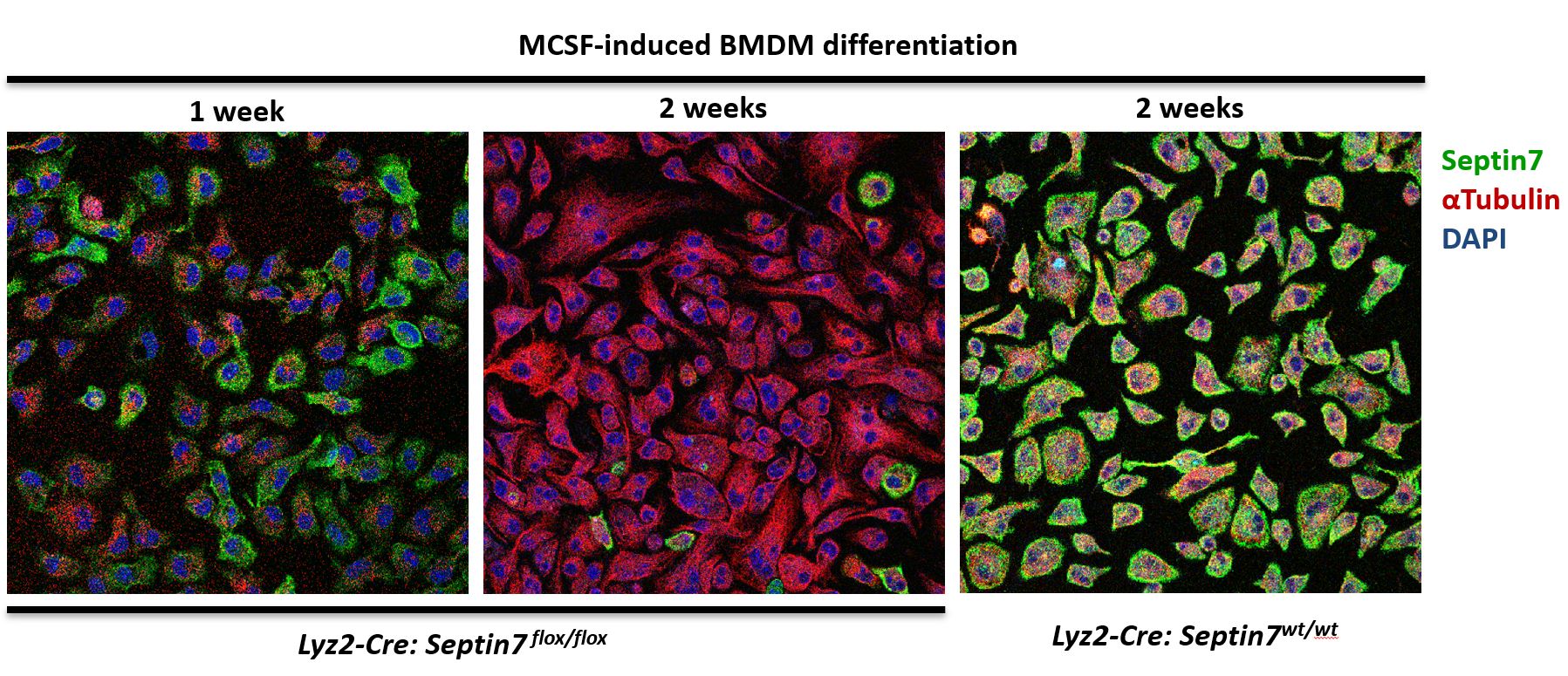

Supplement: Supplementary file 4 [file Image5.JPEG]

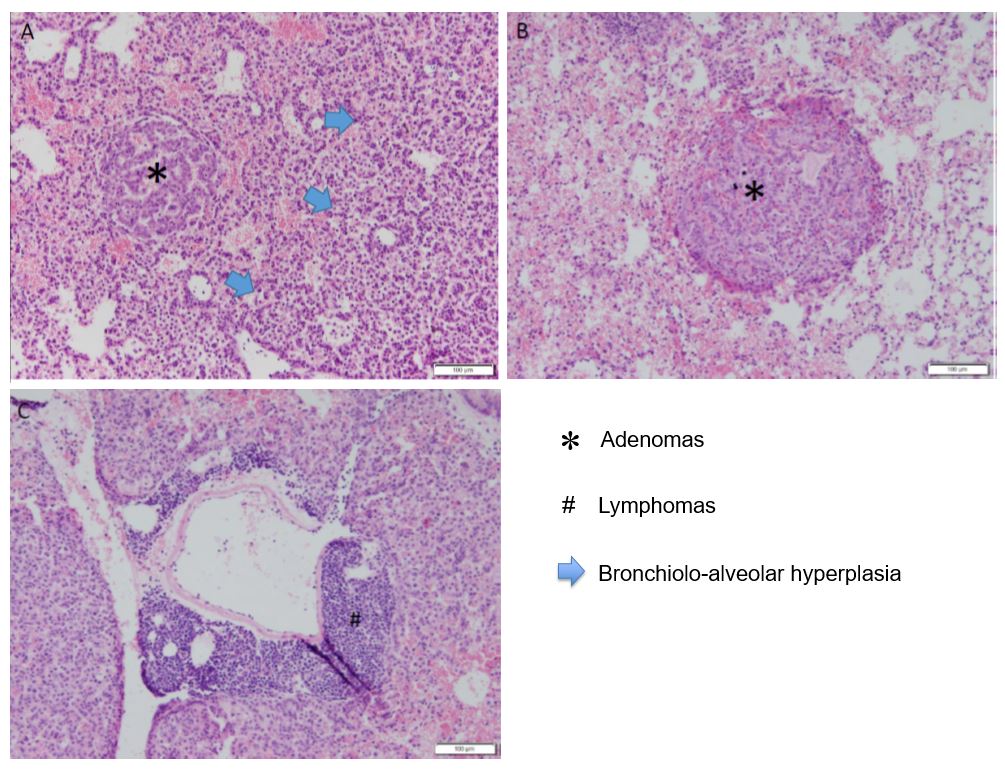

Supplement: Supplementary file 5 [file Image4.jpg]
